# Gut microbiota and therapy for obesity and type 2 diabetes

**DOI:** 10.3389/fendo.2024.1333778

**Published:** 2024-03-26

**Authors:** Luyao Zhang, Pai Wang, Juan Huang, Yanpeng Xing, F. Susan Wong, Jian Suo, Li Wen

**Affiliations:** ^1^ Department of Gastrocolorectal Surgery, General Surgery Center, The First Hospital of Jilin University, Changchun, Jilin, China; ^2^ Section of Endocrinology, Department of Internal Medicine, School of Medicine, Yale University, New Haven, CT, United States; ^3^ National Clinical Research Center for Metabolic Diseases, Key Laboratory of Diabetes Immunology, Central South University, Ministry of Education, Changsha, Hunan, China; ^4^ Department of Metabolism and Endocrinology, The Second Xiangya Hospital, Central South University, Changsha, Hunan, China; ^5^ Division of Infection and Immunity, Cardiff University School of Medicine, Cardiff, United Kingdom

**Keywords:** obesity, type 2 diabetes, gut microbiota, bariatric surgery, pharmacotherapy

## Abstract

There has been a major increase in Type 2 diabetes and obesity in many countries, and this will lead to a global public health crisis, which not only impacts on the quality of life of individuals well but also places a substantial burden on healthcare systems and economies. Obesity is linked to not only to type 2 diabetes but also cardiovascular diseases, musculoskeletal disorders, and certain cancers, also resulting in increased medical costs and diminished quality of life. A number of studies have linked changes in gut in obesity development. Dysbiosis, a deleterious change in gut microbiota composition, leads to altered intestinal permeability, associated with obesity and Type 2 diabetes. Many factors affect the homeostasis of gut microbiota, including diet, genetics, circadian rhythms, medication, probiotics, and antibiotics. In addition, bariatric surgery induces changes in gut microbiota that contributes to the metabolic benefits observed post-surgery. Current obesity management strategies encompass dietary interventions, exercise, pharmacotherapy, and bariatric surgery, with emerging treatments including microbiota-altering approaches showing promising efficacy. While pharmacotherapy has demonstrated significant advancements in recent years, bariatric surgery remains one of the most effective treatments for sustainable weight loss. However, access to this is generally limited to those living with severe obesity. This underscores the need for non-surgical interventions, particularly for adolescents and mildly obese patients. In this comprehensive review, we assess longitudinal alterations in gut microbiota composition and functionality resulting from the two currently most effective anti-obesity treatments: pharmacotherapy and bariatric surgery. Additionally, we highlight the functions of gut microbiota, focusing on specific bacteria, their metabolites, and strategies for modulating gut microbiota to prevent and treat obesity. This review aims to provide insights into the evolving landscape of obesity management and the potential of microbiota-based approaches in addressing this pressing global health challenge.

## 1 Introduction

In the 10th edition of the IDF Diabetes Atlas, an estimated 483 million adults aged 20–79 years worldwide have type 2 diabetes. By 2030, 579 million, and by 2045, 705 million adults aged 20–79 years are projected to be living with type 2 diabetes (1). There is a strong link between type 2 diabetes and overweight and obesity. The WHO defines obesity as a BMI greater than or equal to 30 for adults. Obesity can lead to type 2 diabetes, cardiovascular diseases, musculoskeletal disorders and some cancers, thus increasing medical costs and seriously compromising quality of life in individuals living with obesity (2). It should be noted that BMI definitions of obesity are dependent on ethnicity, which should be taken into account, for example when considering risk of type 2 diabetes and different ranges of weight and BMI relating to obesity and health have been established for different ethnic communities (3, 4). Other measurements such as waist circumference may be a better measure of obesity (5, 6). Obesity is closely related to insulin resistance, glucose intolerance, and changes in various metabolic factors (7). Obesity is also associated with social disadvantage and reduced socio-economic productivity, thus increasingly creating an economic burden (8).

Gut microbiota play an indispensable role in human health (9). Obesity has been associated with perturbation of the intestinal microbiota and changes in intestinal permeability (10). Specifically, diversity and composition of gut microbiota or the presence and abundance of particular bacteria contribute to obesity development (11–14). Gut microbiota are modulated by several factors including diet, genetics, circadian rhythms, anti-obesity medications, probiotics as well as antibiotics (15). Moreover, gut microbiota are also altered by bariatric surgery, leading to microbial changes that may contribute to the observed metabolic benefits after surgery (16, 17). Gut microbiota play a major role in both causing and alleviating obesity; thus, these bacteria should be taken into account when considering effective treatment.

Current treatments of obesity include diet, exercise, pharmacotherapy and bariatric surgery (7, 18). Over the years many different diets have been suggested. One of the healthiest of these in terms of nutritional composition is the Mediterranean diet to tackle obesity and prevent type 2 diabetes (19). Probiotic therapy, brown adipose tissue transplantation and fecal microbiota transplantation also have potential clinical application (20–22). Although there has been significant progress in pharmacotherapy, in recent years (22, 23), with agents that effectively reduce weight and improve metabolism, bariatric surgery is still considered to be the most effective means of sustainable weight loss (24). Currently, most of the anti-obesity medications are contraindicated for use in pregnant women and adolescents (25), and the application of metabolic and bariatric surgery (MBS) in the pediatric population provides evidence-based effective treatment of severe obesity and related comorbid diseases (26, 27). However, bariatric surgery is also associated with significant surgical risks and complications, and thus it is only suitable for the patients with severe obesity with comorbities. Therefore, adolescents and mildly obese patients would benefit from non-surgical treatments to improve their metabolic disorders.

**Figure 1:**
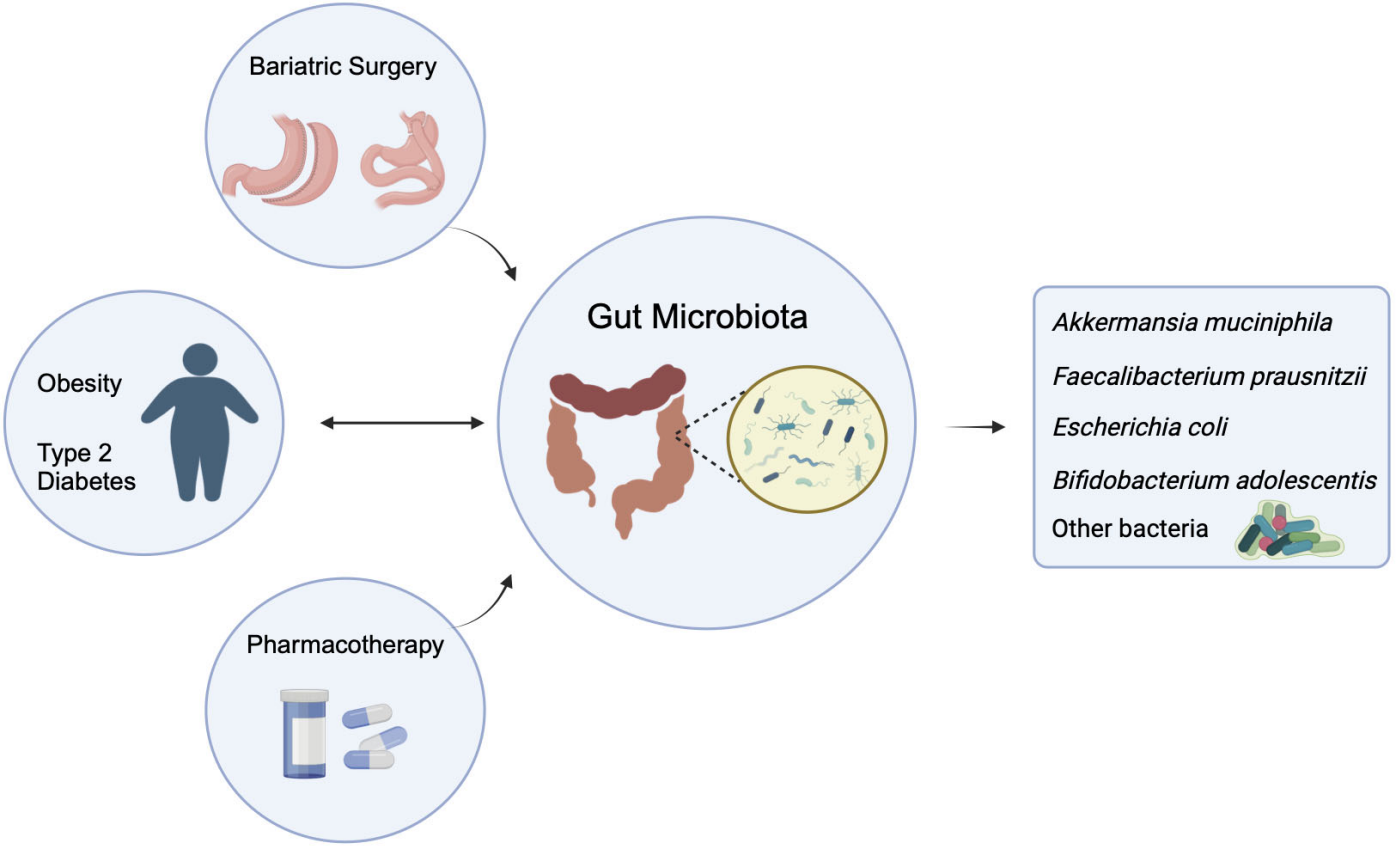
Two-way influence of gut microbiota on obesity and/or type 2 diabetes, and the effect of pharmacotherapy and Bariatric Surgery on the altered gut microbiota. (The figure created by biorender).

In this review, we provide an overview of the studies that have evaluated longitudinal changes in gut microbiota composition and functionality associated with the two currently very effective anti-obesity treatments, pharmacotherapy, and bariatric surgery (7, 23, 28). We also summarize the function of the gut microbiota or defined bacteria, their metabolites, and strategies for modulating gut microbiota for the prevention/treatment of obesity (**Figure 1**).

## 2 The role of intestinal microflora in obesity

In humans there are a vast number of microorganisms, which inhabit the skin, mucous membranes and other surfaces as well as different cavities of the body such as the oral, genitourinary, gastrointestinal, and respiratory tracts. It is estimated that the human microbiota number as many as the number of human cells present in our bodies (29). Intestinal microbiota contain genetic information about 150-fold more than the human gene complement (30). Due to the differences in the tissue environment of different parts of the digestive tract, the microbiota are affected by gastric acid, bile acid and secretions from pancreas, and the distribution of intestinal flora is uneven (31). Gastric flora are relatively sparse with about 10^2^ colony-forming units (CFU)/ml, while the total colonic flora can reach 10^10-12^CFU/ml; moreover, there are obvious differences in the diversity of flora in different segments of digestive tract (32, 33). The intestinal flora of mammals belong mostly to two phyla: Firmicutes and Bacteroidetes, with the other identified phyla including Proteobacteria, Actinobacteria, Fusobacteria and Verrucomicrobia making up a much smaller proportion (34). Previous studies have shown that the gut microbiota play an important role in weight regulation, which can affect fat and energy storage through both energy regulation and gene expression (16). Studies also found that the diversity and abundance of intestinal flora were significantly reduced in people with severe obesity and obesity-related complications, whereas bariatric surgery, carried out as a surgical intervention for morbid obesity, resulted in an increase in microbiota diversity (16). Resveratrol, a natural polyphenol with anti-obesity effect, requires gut microbiota to mediate the effect (35, 36). Those studies suggested that the individuals who are overweight and/or obese may have a specific composition of gut microbiota, which plays an important role in food digestion and energy generation (11, 12, 37).

It is estimated that 10% of the total metabolites in mammalian blood are derived from the gut flora (38), among which short chain fatty acids (SCFAs), lipopolysaccharide (LPS), branched chain amino acids (BCAAs) and bile acids play important roles in metabolic regulation. SCFAs are a product of fermentation of plant polysaccharides produced by different bacteria, which can be absorbed efficiently by the human body (39); The most abundant intestinal SCFAs discovered to date include acetate, propionate, and butyrate, which exhibit anti-inflammatory properties involved in almost every phase of the intestinal mucosal immune response (40). SCFAs not only participate in the regulation of energy metabolism in the body, but also regulate the expression of host genes related to fat metabolism (41). LPS is associated with chronic inflammation, especially in dysbiosis of the intestinal flora with an increase in pathogenic bacteria. These bacteria increase the release of endotoxin LPS, impair the intestinal mucosal barrier and increase intestinal permeability, resulting in low-grade chronic systemic inflammation in the body, which ultimately affects host metabolism, obesity and insulin resistance (42). In addition to LPS, circulating levels of BCAAs tend to be increased in individuals with obesity and are associated with insulin resistance or type 2 diabetes mellitus (43). BCAAS are closely related to the mammalian target of rapamycin signaling pathway (mTOR, mammalian target of rapamycin) in mammals (44), whereas abnormal metabolism of BCAAs may also be associated with accumulation of toxic BCAA metabolites that in turn trigger mitochondrial dysfunction and stress signaling associated with insulin resistance and T2DM (43). Bile acids are metabolites produced by the host and these are biochemically modified by gut bacteria. About 95% of the primary bile acids synthesized in the liver are reabsorbed at the end of the ileum, with half of the remaining 5% bile acids reabsorbed in the colon, and the rest are excreted in feces (45). Bacteria in the small intestine and colon can convert primary bile acids to secondary bile acids, and as a result, the gut microbiota play an important regulatory role in the host’s bile acid cycle. In turn, bile acids influence the composition and overall number of gut microbiota (16, 46). Moreover, reduction in intestinal bile acids contributes to the metabolic benefits of bariatric surgery and a bile acid receptor is required to mediate the effects (47, 48). Other factors are also involved in the interaction between gut microbiota and metabolism, such as Indole derivatives, polyamines, Taurine, ATP, Polysaccharide, hydrogen and methane (40, 49, 50).

## 3 The effects of pharmacotherapy on gut microbiota

Gut microbial composition is highly diverse and continuously modified by endogenous and exogenous factors (51). Besides the factors that affect obesity and diabetes including diet, exercise and lifestyle, various medications, antibiotics being the largest and strongest contributes, can also add to the changes in gut microbiota (52–54). The effects of even short antibiotic exposure can persist and have long term impacts on human intestinal microbiota (55). Other prominent drugs include chronically administered drugs that include anti-diabetic medications, such as metformin, which alter the gut microbiota (56). The U.S. Food and Drug Administration (FDA) has currently approved medications for the treatment of obesity include short-term phentermine, a combination of phentermine/topiramate, orlistat, a combination of naltrexone and bupropion, liraglutide, and semaglutide (22). Phentermine, combined phentermine and topiramate as well as combined naltrexone and bupropion act on the nervous system, but not much research has been done relating to their effects on gut microbiota. Other FDA approved medications for the treatment of type 2 diabetes, among which biguanides, alpha-glucosidase inhibitors (α-GIs), incretin-based drugs, GLP-1 receptor agonists and DPP-4 inhibitors also affect the gut microbiota (56). **Table 1** summaries the effect of pharmacotherapy on gut microbiota (**Table 1**).

**Table 1:** The effect pharmacotherapy on gut microbiota.

Medication	Increase		Decrease	
	Genus/species (phylum)	PMID	Genus/species (phylum)	PMID
**Orlistat**	*Lactobacillus (Firmicutes)*	32328145 (57)	*Alloprevotella (Bacteroidetes)*	34512358 (59)
	*Lactobacillus gasseri (Firmicutes)*	37679239 (58)	*Alistipes (Bacteroidetes)*	36046024 (60)
	*Rhodococcus (Actinobacteria)*	32328145 (57)	*Desulfovibrio (Proteobacteria)*	36046024 (60)
	*Roseburia (Firmicutes)*	32328145 (57)		
**Liraglutide**	*Lactobacillus (Firmicutes)*	27633081 (61) 36643973 (62)	*Roseburia (Firmicutes)*	27633081 (61)
	*Desulfovibrio (Proteobacteria)*	27633081 (61)	*Alistipes (Bacteroidetes)*	33850659 (63) 35095773 (64)
	*Blautia (Firmicutes)*	27633081 (61)	*Proteobacteria (Proteobacteria)*	36643973 (62)
	*Clostridium (Firmicutes)*	33850659 (63)		
	*Parabacteroides (Bacteroidetes)*	36643973 (62)		
	*Akkermansia (Verrucomicrobia)*	33850659 (63) 36643973 (62)		
**Metformin**	*Ruminococcus (Firmicutes)*	34006565 (65)	*Bifidiobacterium (Actinobacteria)*	28470190 (66)
	*Escherichia (Proteobacteria)*	28470190 (66) 34006565 (65)	*Paraprevotella (Bacteroidetes)*	37316616 (67)
	*Lactobacillus (Firmicutes)*	28470190 (66)	*Blautia (Firmicutes)*	37316616 (67)
	*Akkermansia (Verrucomicrobia)*	27999002 (68) 23804561 (69), 25830702 (70) 25038099 (71)	*Faecalibacterium (Firmicutes)*	37316616 (67)
	*Clostridium (Firmicutes)*	25038099 (71)	*Roseburia (Firmicutes)*	34006565 (65)
	*Bacteroides (Bacteroidetes)*	37316616 (67)	*Bacteroides fragilis (Bacteroidetes)*	30397356 (72)
**Acarbose**	*Bifidobacterium (Actinobacteria)*	25327485 (73) 29176714 (74) 34205413 (75)	*Bacteroides (Bacteroidetes)*	29176714 (74) 34205413 (75)
	*Lactobacillus (Firmicutes)*	29176714 (74) 34205413 (75)		
	*Eubacterium (Firmicutes)*	34205413 (75)		
	*Enterococcus faecalis (Firmicutes)*	25327485 (73)		
**Sitagliptin**	*Bifidobacterium (Actinobacteria)*	27631013 (76)	*Blautia (Firmicutes)*	27631013 (76)
	*Lactobacillus (Firmicutes)*	27631013 (76)		
**Dapagliflozin**	*Akkermansia muciniphila (Verrucomicrobia)*	29703207 (77)	*Oscillospira (Firmicutes)*	29703207 (77)
			*Enterococcus (Firmicutes)*	29703207 (77)
**Danagliflozin**	*Alistipes (Bacteroidetes)*	(78)	*Helicobacter (Campylobacterota)*	(78)
	*Alloprevotella (Bacteroidetes)*	(78)	*Mucispirillum (Deferribacterota)*	(78)

### 3.1 Orlistat

Orlistat is a reversible inhibitor of pancreatic and gastric lipase, which has beneficial effects on weight loss and metabolism. Orlistat blocks hydrolysis of triglycerides and reduces absorption of ingested fat by about 30% (79). A study showed that high-fat-diet (HFD) fed mice had enriched intestinal bacteria genera including *Pseudomonas*, *Rhodococcus*, *Roseburia*, and *Acetivibrio* after treatment with orlistat (57). A study in humans showed that the levels of *Lactobacillus* genus and *Lactobacillus gasseri* were significantly higher after 8 weeks of Orlistat treatment (58). Orlistat decreased the relative abundance of *Alistipes*, and *Desulfovibrio* in the fecal microbiota of HFD-fed mice (60). However, other studies did not demonstrate changes in the microbial diversity, predominant bacteria, enterotypes, and fecal SCFAs were not significantly altered by orlistat (59, 80).

### 3.2 GLP-1 receptor agonists

GLP-1 is an incretin hormone secreted by gut L cells, a group of intestinal endocrine cells, in response to food ingestion. GLP-1 has many functions including alterations of gut motility and slowing gastric emptying, inhibiting gastric acid and glucagon secretion but increasing insulin secretion (81). GLP-1 receptor agonists (GLP-1RAs) increased intestinal Bacteroidetes to Firmicutes ratio, decreased obesity-related but increased lean-related microbiota phenotypes (61, 82, 83). Studies in mice treated with liraglutide showed a decrease in *Erysipelotrichaceae Incertae Sedis*, *Marvinbryantia*, *Roseburia*, *Candidatus*, *Arthromitus*, and *Proteobacteria*, which were obesity-related phylotypes and an increase in *Akkermansia*, *Blautia*, *Lactobacillus Parabacteroides* and *Coprococcus*, which were lean-related phylotypes (61, 62), with *Akkermansia muciniphila* contributing to intestinal health and glucose homeostasis (84). In studies in humans, the genera *Collinsella*, *Akkermansia* and *Clostridium* were enriched in the post-liraglutide-treatment group whereas *Alistipes* was decreased (63, 64). Little is known about the effect of semaglutide, another GLP-1 RA, on the composition of gut microbiota, although it is highly efficacious in the treatment of obesity (22).

### 3.3 Metformin

Among all the anti-diabetic medications, metformin, the first-line of medication for T2D treatment, has been most studied in relation to interaction with the gut microbiota. The therapeutic mechanism of metformin, at least in part, requires the involvement of intestinal microbiota (85). Metformin increases a number of different SCFA-producing bacteria, including *Blautia, Butyrivibrio, Bifidobacterium bifidum, Megasphaera*, and *Prevotella* (68, 86). Metformin is also associated with an increase in *Akkermansia muciniphila*, a mucin degrading microbiota (68). In studies in humans, the genera *Akkermansia*, *Ruminococcus*, *Escherichia* and *Lactobacillus* were enriched whereas *Bifidiobacterium*, *Paraprevotella*, *Blautia* and *Faecalibacterium* decreased in the post-Metformin-treatment group (65–67). However, these are not the only associated bacteria, and another study in humans suggested that the improvement of metabolism through metformin was mediated by a *Bacteroides fragilis* (72). The increase of *Akkermansia* post Metformin treatment is prominent and has been found in both human and mouse model studies (69–71).

### 3.4 Alpha glucosidase inhibitors

Acarbose, an alpha glucosidase inhibitor used to treat T2DM, is derived from bacterial fermentation; thus, it is logical that acarbose is likely to affect the gut microbiota (87). It is less used with the advent of newer medications as individuals find it difficult to take because of flatulence. Acarbose increases the relative abundances of *Lactobacillus* and *Bifidobacterium* in the gut microbiota and depletes Bacteroides in patients with type 2 diabetes (73–75).

### 3.5 Other anti-diabetic drugs

Sitagliptin, a dipeptidyl peptidase (DPP|0-4) inhibitor, increases the relative abundance of Bacteroidetes and Proteobacteria but decreases Firmicutes (88). In an animal study, the authors found that sitagliptin affects the abundance of SCFA-producing bacteria, *Blautia*, *Roseburia* and *Clostridium*, as well as probiotics *Lactobacillus*, *Bifidobacterium* (76). Also in animal studies, dapagliflozin and canagliflozin, SGLT-2 inhibitors, were found to increase the ratio of Bacteroidetes to Firmicutes. Moreover, dapagliflozin increased the abundance of *Akkermansia muciniphila* and decreased *Oscillospira* whereas canagliflozin increased the abundance of *Olsenella*, *Alistipes* and *Alloprevotella*, but decreased the abundance of *Helicobacter* and Mucispirillum (77, 78).

## 4 The effects of bariatric surgery on gut microbiota

Although diet, exercise and pharmacotherapy can effectively reduce an individual’s body weight and improve their metabolism, metabolic/bariatric surgery is still considered to be the most effective mean of sustainable weight loss (24, 89). The American Society for Metabolic and Bariatric Surgery (ASMBS) endorsed common procedures including Adjustable Gastric Band (AGB), Sleeve Gastrectomy, Roux-en-Y Gastric Bypass (RYGB), Biliopancreatic Diversion with Duodenal Switch (BPD/DS), and Single Anastomosis Duodeno-Ileal Bypass with Sleeve Gastrectomy (SADI-S), although AGB is less successful as a treatment for type 2 diabetes (90). Bariatric surgery is generally reserved for patients with severe obesity (BMI > 40 kg/m2) and moderate obesity (BMI > 35 kg/m2) with at least one additional serious complication such as hypertension, diabetes, or sleep apnea syndrome (91). If other interventions for treating obesity and obesity-related co-morbidities have not been effective, bariatric surgery should be considered for the individuals with class I obesity (BMI 30–34.9 kg/m2). It is noteworthy that BMI risk zones should be adjusted to define obesity with a BMI threshold of 25–27.5 kg/m^2^ in Asian population (89). The American Society for Metabolic and Bariatric Surgery Pediatric Committee and the American Academy of Pediatrics recommend that children/adolescents with class II obesity (BMI=35 to 39.9) and a co-morbidity, or with class III obesity (BMI=40 and greater) should be considered for bariatric surgery (92, 93). Bariatric surgery improves blood glucose, increases insulin sensitivity, promotes insulin secretion, and changes the levels of incretin (94). In addition, researchers believe that the efficacy of bariatric surgery is also closely related to changes in both the type and abundance of gut microbiota. The surgery effect can be sustained for ten years, allowing patients to achieve sustained weight loss (95).

Previous studies have found that the impact of bariatric surgery on gut microbiota were reflected in the following aspects. Firstly, bariatric surgery alters the number of gut microbiota. Zhang and colleagues were pioneers who found changes of intestinal flora in bariatric surgery patients in 2009 and reported that Firmicutes were significantly decreased in post-gastric-bypass individuals (96). In the following year, Furet and co-authors observed that RYGB rapidly induced gut microbiota adaptation, through which the *F. prausnitzii* species was directly linked to reduction in the low-grade inflammatory state in obesity and diabetes that was independent of calorie intake (97). Secondly, bariatric surgery affects the gut microbiota by changing the patient’s eating habits. Individuals who underwent gastric bypass surgery had less interest in high-fat and high-sugar foods after surgery (98, 99), and these changes in dietary composition and amount of the food intake also altered the levels of Firmicutes and Bacteroides in the intestinal flora (100). Thirdly, bariatric surgery also changes the pH of the digestive tract as the surgery changes the structure of the gastrointestinal tract, which affects the secretion of gastric acid and the pH of the gastrointestinal tract, resulting in changes in the intestinal luminal environment and concomitantly, the intestinal flora (101, 102). Moreover, changes in colonic pH can affect the composition of colonic microbial communities as well as the proportion of SCFA-producing bacteria (103). Fourthly, bariatric surgery changes enterohepatic cycle diversion that affects gut microbiota. Gastric bypass surgery shortens the time for food emptying and can lead to changes in both primary and secondary bile acid levels, both of which have antimicrobial properties (104, 105). The administration of secondary bile acids altered the composition of the gut microbiota with increased Firmicutes and decreased Bacteroidetes levels (106). Fifthly, the use of antibiotics for bowel preparation for surgery. Bariatric surgery requires the use of prophylactic antibiotics, and different types of antibiotics have different effects on the growth of intestinal bacteria and the overall diversity of bacteria (107). Claesson et al. found that the use of antibiotics increased the abundance of Bacteroides and decreased the proportion of Firmicutes (108). Lastly, gastric bypass surgery can lead to changes in neurohormones which include increased secretion of GLP-1 (109), known to signal satiety, reduce food intake, decrease stomach motility and increase β-cell insulin production (16). All these factors may have an impact on the intestinal microbiota. In addition, GLP-1 directly regulates the composition and function of the intestinal flora (110).

Overall, bariatric surgery changes the internal environment of the intestine and the secretion of gastrointestinal hormones by altering the structure of the gastrointestinal tract that causes the amount and the type of food intake, thereby improving glycolipid metabolism and insulin resistance. All these factors assist in achieving the sustained weight loss that is closely related to the changes in intestinal flora after surgery. Studies using modern technologies, which include large-scale high-throughput sequencing, metagenomic sequencing and metabolomics will provide us with better and broader understanding of the changes in the composition and function of intestinal flora after surgery. This knowledge will help us to identify specific bacteria strains and/or related metabolites for potentially more effective treatment of obesity and associated complications. **Table 2** summarizes the alterations in gut microbiota post-surgery (**Table 2**); further details are reviewed below.

**Table 2:** The effect of bariatric surgery on gut microbiota.

Surgery type	Increase		Decrease	
	Genus/species (phylum)	PMID	Genus/species (phylum)	PMID
**AGB**	*(Actinobacteria)*	31256356 (111)		
**SG**	*Bulleidia (Firmicutes)*	28570438 (112) 28649469 (113)	*Coprococcus comes (Firmicutes)*	25710027 (114)
	*Roseburia intestinalis (Firmicutes)*	27738970 (115)	*Blautia (Firmicutes)*	36008102 (116)
	*Faecalibacterium prausnitzii (Firmicutes)*	25710027 (114)		
	*Parabacteroides (Bacteroidetes)*	36008102 (116)		
**RYGB**	*Akkermansia muciniphila (Verrucomicrobia)*	37196633 (117) 19164560 (96) 27306058 (118)	*Lactobacillus (Firmicutes)*	20876719 (97) 23719559 (119)
	*Faecalibacterium prausnitzii (Firmicutes)*	20876719 (97) 23032991 (120)	*Bifidiobacterium (Actinobacteria)*	20876719 (97) 23719559 (119)
	*Coprococcus comes (Firmicutes)*	23032991 (120)		
	*Roseburia intestinalis (Firmicutes)*	27738970 (115)		
	*Escherichia coli (Proteobacteria)*	27306058 (118) 20876719 (97) 26244932 (95)		
	*Blautia (Firmicutes)*	37196633 (117)		
	*Prevotella (Bacteroides)*	20876719 (97)		
**BPD/DS**	*Parabacteroides (Bacteroidetes)*	36008102 (116)	*Blautia (Firmicutes)*	36008102 (116)
	*Bifidobacteriales (Actinobacteria)*	31165976 (121)		
**SAID-S**	*Sutterella (Proteobacteria)*	34686781 (122)	*Ruminococcus (Firmicutes)*	34686781 (122)
	*Clostridium (Firmicutes)*	34686781 (122)	*Oscillibacter (Firmicutes)*	34686781 (122)

AGB, Adjustable Gastric Band; SG, sleeve gastrectomy; RYGB, Roux-en-Y gastric bypass; BPD/DS, Biliopancreatic Diversion with Duodenal Switch; SADI-S, Single Anastomosis Duodeno-Ileal Bypass with Sleeve Gastrectomy.

### 4.1 Adjustable gastric band

AGB surgery is a gastric restrictive procedure in which a prosthetic band is placed around the upper stomach to compartmentalize it into a small pouch and a large remnant, and the adjustable band allows changes in the stoma size. Although AGB surgery has been reported to increase gut microbial gene abundance (123) and one study showed that *Actinobacteria* increased post AGB surgery (111), more in depth effects of the AGB procedure on gut microbiota have not been investigated as much as in bariatric surgery (124). This is partly because AGB does not make radical changes in gastrointestinal anatomy (125).

### 4.2 Sleeve gastrectomy

Sleeve gastrectomy is another gastric restrictive procedure that involves permanently removing approximately 80% of the stomach. The remaining stomach retains the pylorus with a shape like a banana. Sleeve gastrectomy significantly alters gut microbiota. *Bacteroides Thetaiotaomicron*, an obesity-associated gut microbial species, was markedly decreased in obese individuals after sleeve gastrectomy and this procedure also alleviated diet-induced body-weight gain and adiposity in mice (11). In addition, *Roseburia*, which are highly correlated with T2DM, were also affected by sleeve gastrectomy (126, 127). In a mouse model study, *Roseburia* were found to be negatively correlated with AUC in the glucose tolerance test after sleeve gastrectomy (128). Multivariate analysis demonstrated differential abundance of *Bulleidia* increased after surgery (112, 113). SG resulted in increased Bacteroidetes phyla, and an increase in *Roseburia* species was observed among those achieving diabetes remission (115). Another study showed that, except for a postoperative increase in *Faecalibacterium prausnitzii*, laparoscopic sleeve gastrectomy (LSG) resulted in the postoperative reduction in Firmicutes including *Coprococcus*, a butyrate-producing bacterium (114).

### 4.3 Roux-en-Y gastric bypass

RYGB is both a restrictive and malabsorptive procedure in which a small gastric pouch (∼30 ml) is created and directly linked to the distal jejunum by the Roux limb. The proximal part of the jejunum is subsequently anastomosed 1.5 m below the gastrojejunal anastomosis. It is one of the most common and highly effective operations for treating morbid obesity with or without T2DM. Interestingly, the gut microbiota richness was increased after RYGB (119). Firmicutes were significantly decreased whereas Bacteroidetes were significantly increased in the individuals post-RYGB. Moreover, *Akkermansia* became prominent in post-gastric-bypass individuals, comparable to the individuals with normal body weight (96, 117, 118). The abundance of *F. prausnitzii*, which was directly correlated to fasting blood glucose, was lower in individuals with morbid obesity with or without diabetes before RYGB, but increased three months post-RYGB and remained stable until the study end point - six months post-RYGB (97, 120). It was demonstrated that the altered human microbiota due to the surgery reduced fat deposition in the germ-free recipient mice colonized with bacteria from stool samples from the human donors (95).

### 4.4 Biliopancreatic diversion with duodenal switch

The BPD-DS probably is the most effective approved metabolic operation for the treatment of obesity with T2DM. However, BPD is rarely performed today due to the metabolic disturbances and nutritional deficiencies that occur after the procedure (129). There are two steps of the procedure – the first is to create a sleeve-like stomach, after which the distal jejunum is connected to the outlet of the newly-created stomach. Germ-free mice colonized with microbiota from patients post-BPD-DS, had lower villus height/width and crypt depth in the distal jejunum, as well as lower intestinal glucose absorption (116). The colonized mice also had higher *Parabacteroides* and lower *Blautia*, which coincided with improved blood glucose (116). Studies in Wistar Rats showed that the rats had a higher abundance of *Bifidobacterium* and lower abundance of the *Clostridiales* in the common and alimentary limbs, similar to that in fecal samples, taken after BPD-DS. Moreover, both the body weight gain and fat mass were positively correlated with the microbial abundances of *Clostridiales*, contributing to the positive metabolic outcomes of BPD-DS (121, 122).

### 4.5 Single anastomosis duodeno-ileal bypass with sleeve gastrectomy

SADI-S is the latest procedure endorsed by ASMBS. SADI-S is similar to the BPD-DS but simpler than BPD-DS and RYGB, as there is only one anastomosis. It is an excellent option for patients who have already had a sleeve gastrectomy and are seeking further weight loss. The study using Wistar rats showed that the altered gut microbiota post-SADI-S shared similar signatures and changes in metabolism as seen in the other two types of restrictive and malabsorptive procedures, RYGB and BPD/DS (122).

## 5 Bacteria closely related to obesity and diabetes

How can we define a causal role for a bacterium in diabetes or obesity? The first criterion of Koch’s postulates is to demonstrate a putative agent causes an infectious disease (130). Evans proposed a modified version as a unified concept for establishing causation by a putative cause of disease. In the modified postulates, three lines of evidence are required: a) an association between the disease phenotypes and the presence of the cells or genetic material of the putative cause; b) the reproduction or reduction of the disease phenotypes by experimental addition or removal of the putative cause in humans or animals; c) and the occurrence of host molecular responses that mechanistically connect the presence of the putative cause to the occurrence of the disease (9, 130). Here, we will use the modified version of Koch’s postulates as a conceptual framework for organizing evidence to illustrate the causative role of gut microbiota in obesity and diabetes, as summarized in **Table 3** (**Table 3**).

**Table 3:** Summary of the altered gut microbiota in obesity and T2D and the outcome after medical intervention.

Genus/species (phylum)	Obesity Type 2 diabetes	Pharmacotherapy	Bariatric Surgery	Role of microorganisms
*Akkermansia muciniphila (Verrucomicrobia)*	**↓**	**↑**	**↑**	influence gut permeability through the regulation of tight junctions
*Faecalibacterium prausnitzii (Firmicutes)*	**↓**	**↑**	**↑**	increases insulin sensitivity produces butyrate
*Escherichia coli (Proteobacteria)*	**↑↓**	**↑**	**↑**	The role of E. coli in obesity and/or type 2 diabetes is debatable.
*Bifidobacterium Adolescentis (Actinobacteria)*	**↓**	**↑**	**↑**	increase insulin sensitivity

**↑**: increased abundance of microorganisms; **↓**;decreased abundance of microorganisms.

### 5.1 Akkermansia muciniphila

*Akkermansia muciniphila*, of the phylum *Verrucomicrobia*, is extensively present in the intestinal mucosa of humans and other mammals (131), and they account for 1 to 3 percent of the total gut microbiota in healthy adults (132). *A. muciniphila* has a number of beneficial features in respect of metabolism and is inversely correlated with fasting glucose, waist-to-hip ratio and subcutaneous adipocyte diameter, and importantly, negatively correlated with diabetes, obesity, and metabolic syndromes (133, 134). Oral administration of *A. muciniphila* at a dose of 2 × 10^8^ bacterial cells per day reversed HFD-induced obesity in mice, and improved several metabolic parameters in both mice and humans (69, 135, 136). *A. muciniphila* has also been shown to be important in changes associated with therapy used for diabetes. HFD-fed mice treated with metformin showed a higher abundance of *Akkermansia* than HFD-fed mice without metformin treatment (69). In people living with type 2 diabetes, increased abundance of *A. muciniphila* was seen in those treated GLP1-agonist therapy (137). Surgical treatment with RYGB also led to increased relative abundance of *A. muciniphila* (118). With respect to physiological changes associated with *A. muciniphila*, the bacteria strongly adhere to human colonic cell lines and improve enterocyte monolayer integrity (138). *A. muciniphila* also induces interleukin 8 (IL-8) production by enterocytes (138), the role of which is not clear. *A. muciniphila*-derived extracellular vesicles influence gut permeability through the regulation of tight junctions (139). A study also showed that Pili-like proteins of *A. muciniphila* modulate host immune responses and gut barrier function (140). Thus, according to the modified version of Koch’s postulates, the above examples are evidence for the role of *A. muciniphila* with a reduction in the bacteria occurring in obesity and diabetes.

### 5.2 Faecalibacterium prausnitzii

*Faecalibacterium prausnitzii* (*F. prausnitzii*) is one of the most abundant bacterial species in the colon of healthy human adults, representing more than 5% of the total intestinal bacterial population (141). This is another bacterial species that is associated with improved glucose metabolism and reduced inflammation. People living with obesity have lower levels of *F. prausnitzii* in their gut microbiota (142, 143). *F. prausnitzii* transplantation has been shown to be an effective therapeutic approach for diabetes and related complications. The procedure prevents low-grade inflammation due to altered gut microbiota, protects the pancreas from autoimmunity and increases insulin sensitivity (144, 145). The abundance of *F. prausnitzii* was increased in people living with type 2 diabetics who had GLP1-agonist therapy (137). The level of *F. prausnitzii*, which was lower in the individuals living with Type 2 diabetes before RYGB, increased at 3-months post-surgery and remained stable at 6-months post-surgery (97). *F. prausnitzii* produces butyrate, which beneficially modulates the intestinal immune system, oxidative stress and colonocyte metabolism (146, 147). Taken together, the above studies demonstrated the pathogenic role of *F. prausnitzii* reduction in obesity and diabetes, based on the modified version of the Koch’s postulates.

### 5.3 Escherichia coli

*Escherichia coli* (*E. coli*) is the most abundant commensal aero-anaerobic Gram-negative bacillus of the vertebrate gut, and most *E. coli* strains are harmless (148). However, *E. coli* have been linked to increased weight in both humans and in mice. *E. coli* was found to be predictive of the development of insulin resistance in postmenopausal obese Caucasian females in Sweden (149). In a HFD-fed mouse model of obesity, *E. coli* significantly increased body weight and adiposity, and induced impaired glucose tolerance (150). Therapeutically, in a human randomized trial, paradoxically, the investigators found that metformin treatment significantly increased amplicon sequence of *E. coli* variants (65). The increase of the relative abundance in *E. coli*, was also observed in patients after VGB or RYGB (95, 97). However, colonization of *E. coli* in germ-free mice led to increased inflammation in liver, adipose tissue and intestinal tissue under a HFD regimen (150). The role of *E. coli* in obesity and/or type 2 diabetes is debatable as there are contradictory findings; thus, we need to further clarify the mechanism of action of *E. coli* in obesity and diabetes in future research.

### 5.4 Bifidobacterium adolescentis

Bifidobacteria are gram-positive and one of the most important bacteria in the human and animal intestinal environment. The gut microflora in people living with T2D had a lower abundance of *Bifidobacterium adolescents* (151) whereas metformin promoted the growth of *Bifidobacterium adolescentis* (152). In an obese rat study, *Bifidobacterium adolescentis* was increased significantly after RYGB (153). Moreover, *Bifidobacterium adolescentis* supplementation ameliorated visceral fat accumulation and insulin sensitivity of HFD-induced obesity in rats (154). *B. adolescentis* has a higher number of core genes including unique environmental tolerance, carbon and nitrogen utilization genes, and a blood sugar regulation gene which was likely the reason why *B. adolescentis* may reduce T2D, shown in a mouse study (155). Thus, according to the concept of modified version of Koch’s postulates, a reduction in *B. adolescentis* could play a causative role in obesity and diabetes.

## 6 Conclusions and perspectives

There has been substantial evidence supporting the role of gut microbiota in the development of obesity and related diseases. It is clear that the composition of the gut microbiota evolves after drug treatment or bariatric surgery, but these observations remain to be confirmed in larger and longer-term cohorts (especially in the patients who regained weight). Although the ratio of Firmicutes/Bacteroidetes was reduced by dietary and surgical interventions, in our review of the literature, we have highlighted an increase of Firmicutes abundance at genus/species levels in almost all types of interventions. Moreover, there are more genera associated with *Lactobacillus* and fewer with *Ruminococcus*, both of which are Firmicutes phyla. Interestingly, *Akkermansia muciniphila*, phylum Verrucomicrobia, was also increased after the interventions and it is worth mentioning that *Akkermansia muciniphila* has been reported to be useful in a new generation of probiotics. It would be helpful in the clinical setting if some individual bacterial genera/species/strains could serve as biomarkers for evaluation of diabetes and the efficacy of treatment rather than the Firmicutes/Bacteroidetes ratio or microbial diversity index. It is clear that in studies of gut microbiota associated with metabolic disorders, a deeper investigation of bacterial differences at the strain level will be required, as well as understanding changes in other microorganisms including enteroviruses (156) and intestinal fungi (157). It is important that there are more adequately designed studies using next-generation sequencing and ‘omics’ technologies to identify the bona fide causal microorganisms, which could lead to more specific interventions. However, that does not exclude research based on Koch’s hypothesis to identify specific gut bacteria that may play a pathogenic role in obesity and diabetes, or microbes which may be decreased in these conditions, which may lead to novel opportunities for treatment of obesity and diabetes prevention and treatment. Interventions that reduce species associated with uncontrolled diabetes but increase species associated with a healthy gut of individuals without diabetes would be ideal for treating diabetes.

There is no doubt that bariatric surgery affects the gut microbiota, altering their composition and function. Bariatric surgery changes the anatomy of digestive tract, affects gut hormonal homeostasis, influences the amount and choice of food ingestion, all of which contribute to the alteration of the microbiota composition. This complexity is challenging for clinical research. Next-generation sequencing and ‘omics’ technologies will provide powerful tools for better understanding of how the gut microbiota and host metabolism interact and influence the outcome of bariatric surgery. There are still some inconsistent results from studies investigating whether altered gut microbiota cause weight loss after bariatric surgery for the treatment of obesity and diabetes. Thus, whether microbiota evolution is a cause or a consequence of weight loss and improvements in obesity-related diseases is still inconclusive. Much research is needed in investigating the role of the gut microbiota in metabolic outcomes after bariatric surgery and the underlying mechanisms that mediate this vital relationship, in which Koch’s hypotheses may aid our understanding. However, healthy gut microbiota will help better therapeutic outcomes of the surgery and the gut microbiota could be a biomarker for predicting the efficacy of the bariatric surgery and potentially a useful tool for personalized medicine.

We have little knowledge about the majority of gut bacteria, although high throughput sequence technology has helped us identify some of these. Moreover, most gut bacteria cannot be cultured due to inflexible nutrient requirements and/or the need to co-exist with other bacteria (158). However, the ultimate proof of the causality of gut bacteria in obesity will be their ability to induce obesity in a non-obese host, such as germ-free mice (50) as well as the proof of ameliorating obesity when removing them. Although neither humans nor animals live in a germ-free environment, the gnotobiotic animal model provides unique opportunities to better understand the function of the gut bacteria and the molecular interactions between obesity-inducing bacteria and the host. In addition to the known host responses to endotoxins, transcriptomic and proteomic analyses are powerful assets to help us better understand the genome-wide responses of different host tissues to the colonized “pathogens”. With improved knowledge, we will be able to identify potential new targets and design more effective pharmacological interventions. With the increased need for organoid research, highly functional intestinal organ culture systems based on microfluidics have been developed (159). The authors found that observations made previously *in vivo* could be reliably replicated *in vitro* with the organoid system, while being able to extract compelling new insights due to the tightly controlled nature of the organoid system (159). New systems like this will be critical for elucidation of the more complex connections between host and microbiome.

The gut microbiota plays an integral role in human health and studies in this area have opened new areas of research in basic human biology and clinical medicine. Koch’s hypothesis can be applied and there is some evidence that specific gut bacteria may play a causative role in non-communicable diseases such as obesity. The study of the gut microbiota has led and will in the future further advance the development of novel means to combat various health issues including the epidemic of obesity and associated devastating complications, that increasingly challenge human society.

## Author contributions

LZ: Writing – original draft. PW: Writing – original draft. JH: Writing – original draft. YX: Writing – original draft. FW: Writing – review & editing. JS: Supervision, Writing – review & editing. LW: Supervision, Writing – review & editing.
